# Effectiveness of a Computerized Home-Based Cognitive Stimulation Program for Treating Cancer-Related Cognitive Impairment

**DOI:** 10.3390/ijerph20064953

**Published:** 2023-03-11

**Authors:** Jose L. Tapia, María Teresa Taberner-Bonastre, David Collado-Martínez, Athanasios Pouptsis, Martín Núñez-Abad, Jon Andoni Duñabeitia

**Affiliations:** 1 Centro de Investigación Nebrija en Cognición (CINC), Universidad Nebrija, 28015 Madrid, Spain; 2 Servicio de Oncología Médica, Hospital Universitario de la Ribera, 46600 Valencia, Spain; 3 AcqVA Aurora Center, The Arctic University of Norway, 9019 Tromsø, Norway

**Keywords:** cancer, cognitive decline, chemo-fog, chemo-brain, oncology, cognitive intervention, computerized cognitive stimulation

## Abstract

Cancer patients assert that after chemotherapy their cognitive abilities have deteriorated. Cognitive stimulation is the clinical treatment of choice for reversing cognitive decline. The current study describes a computerized home-based cognitive stimulation program in patients who survived breast cancer. It aims to assess safety and effectiveness of cognitive stimulation in the oncology population. A series of 45-min training sessions was completed by the participants. A thorough assessment was performed both before and after the intervention. The mini-Mental Adjustment to Cancer Scale, the Cognitive Assessment for Chemo Fog Research, and the Functionality Assessment Instrument in Cancer Treatment–Cognitive Function served as the main assessment tools. The State-Trait Anxiety Inventory, Beck Depression Inventory, Brief Fatigue Inventory, and Measuring Quality of Life–The World Health Organization data were gathered as secondary outcomes. Home-based cognitive stimulation demonstrated beneficial effects in the oncology population, with no side effects being reported. Cognitive, physical, and emotional improvements were observed, along with decreased interference in daily life activities and a better overall quality of life.

## 1. Introduction

Up to 80% of patients who undergo cancer surgery complement their intervention with adjuvant therapies, including chemotherapy, radiotherapy, targeted therapy, biological therapy, and hormone therapy [1,2,3,4,5,6]. These therapies aim to prevent recurrence or growth of cancer cells left after surgery, thereby increasing the survival rate [7]. Educational campaigns promoting lifestyle changes and screening tests have also contributed to reducing cancer mortality rates [8,9], yielding better outcomes for patients [10]. The current estimate for 5-year relative survival rate following a cancer diagnosis is around 70%, a number that noticeably increases when breast cancer is specifically taken into account [11]. Despite these encouraging statistics, it has been noted that the rise in survivability is accompanied by several sequelae and unfavorable outcomes sometimes associated with the adjuvant therapies [12,13,14,15,16]. The side effects vary depending on the specific treatment, duration, frequency, and individual patient’s health and medical history. For instance, common side effects include nausea and vomiting, fatigue, diarrhea, appetite loss or mood swings [17]. Long-term adversities may include high blood pressure, joint pain, hormonal imbalances, and damage to organs such as the heart, liver, lungs, or kidneys [18,19,20]. Independently to the aforementioned side effects, cancer survivors frequently experience cognitive impairment, occurring in up to 80% of patients at some point [21,22,23]. Various experimental trials have demonstrated that chemotherapeutic agents, present in adjuvant therapies, may induce central nervous system toxicity negatively affecting cognitive skills reliant on the hippocampus and frontal lobes [24,25,26]. Thus, difficulties experienced by cancer patients related to the ability to pay attention, concentrate, learn, reason, process, use executive functions, and be spatially aware, are likely to be due to such neurotoxicity [27,28,29,30,31].

Being among the most commonly diagnosed cancers [32] breast cancer is in the spotlight for developing cognitive impairment due to several factors. With the very high survival rate [11] many more women are living long enough after their cancer diagnosis to experience the long-term effects of their treatment, and breast cancer is often treated with chemotherapy, which has been shown to be a risk factor for cognitive impairment [33]. In addition, because of its characteristics, breast cancer patients often present less secondary symptomatology than other types of cancer. In lung cancer, for example, respiratory symptoms such as cough, chest pain, and shortness of breath can affect patients’ quality of life and may overshadow cognitive symptoms [34,35]. On the other hand, breast cancer patients may have fewer physical symptoms that distract from cognitive impairment, making it a priority symptom that requires attention [36,37,38].

The subjective cognitive complaints reported by breast cancer patients relate to challenges in a variety of areas, such as retrieving words or remembering names, maintaining concentration, conversation fluency, multitasking, planning, and organizing tasks, or feeling less alert or mentally exhausted at the end of the day [22,39,40,41]. In addition to the subjective complaints, recent studies have corroborated these impairments through neuropsychological assessments, finding below-average scores in attention, short-term memory, executive function, processing speed, concentration, and visuospatial ability [42,43]. About 40% of cancer patients present cancer-related cognitive impairment (CRCI) right after diagnosis, most probably due to the shock-related emotional impact [44], and this percentage rises to 75% during chemotherapy treatment, and to 60% months or even decades after successfully concluding their treatment [13,14,15,16,17,18]. As a consequence of this cognitive dysfunction, patients gradually experience a decrease in their capacity to carry out everyday duties and may represent an additional burden for reintegration into the workplace and getting back into social and family routines, therefore, suffering an impact in their life quality [40,45,46,47,48], which can lead to early retirements, work absences, extended vacations, and financial worries [49,50].

Despite frequent patient complaints, the scarcity of neuropsychologists and the lack of available information on cognitive assessment and intervention lead physicians to neglect cancer-related cognitive impairments [40,51]. Most clinical practice guidelines in oncology address aspects such as physical functioning, mood disturbances, or sleep problems [52]. However, it was not until the very latest update of the Clinical Practice Guidelines in Oncology published by the National Comprehensive Cancer Network (NCCN), that a systematic assessment of cognitive function by self-report has been included as one of the general principles, but there is still little evidence regarding effective intervention in this regard [53,54]. Therefore, the need for interventional research targeting CRCI remains an open matter.

Neural plasticity, understood as the brain’s ability to reorganize its structure and functions through changes in the strength and number of synapses in response to internal or external stimuli [55,56,57,58], is closely linked to brain-derived neurotrophic factor (BDNF) and cognitive function [59]. Cognitive stimulation and training can increase BDNF levels, which is associated with better cognitive function, particularly in executive functions and memory [60,61,62,63]. In addition to BDNF, 17β-estradiol (E2) has been shown to play a critical role in cognitive function, specifically in hippocampal-dependent cognitive functions such as spatial learning and memory [64]. The widespread expression of aromatase in neurons in the human hippocampus and cortex allows for the local production of E2 [65]. However, systemic aromatase inhibitor treatment for advanced breast cancer can lead to E2 deficiency, resulting in impaired hippocampal-dependent memory and decreased hippocampal activity during encoding [66,67]. Therefore, interventions that focus on cognitive functions, such as executive function and memory, may be effective in inducing BDNF and enhancing neural plasticity to improve cognitive function in individuals with cognitive decline due to cancer treatment and E2 deficiency [68]. Advances in the understanding of this neural functioning have led to the development of a range of therapeutic applications in the form of cognitive stimulation interventions, which aim to improve brain function by increasing the strength and efficiency of neural connections [69,70,71,72]. These interventions can take the form of computer-based training programs or cognitive rehabilitation, and have been shown to be effective in improving cognitive function in individuals with a range of neurological and psychiatric disorders [73,74,75,76,77,78,79]. Therefore, considering the promising results from other diseases that are also linked to cognitive dysfunction, non-pharmacological intervention approaches based on cognitive stimulation represent a potential solution [80]. Thus, being considered as the enhancement of a cognitive skill through practice, cognitive stimulation programs include repetitive and standardized tasks, oriented to specific cognitive domains, and can be easily self-administered by the patient without the need for professional supervision, with an acceptable rate of dropouts [74,81]. In clinical settings, cognitive stimulation has been proven effective in numerous populations, with reports of cognitive improvement in patients with mood alterations [76], psychiatric disorders [82], neurodegenerative conditions [83], stroke [84], eating disorders [85], or sleep disturbances [74,86]. In this line, it is worth noting that the promising results of a first pilot study of cognitive stimulation aimed at patients with CRCI already suggested benefits in executive functions, information processing speed, and memory after intervention [87]. Several subsequent studies have corroborated these results, finding further improvements in general cognitive functioning, speech fluency, and visuospatial skills in cancer patients following cognitive stimulation [88].

Additionally, it has been shown that computerized cognitive stimulation programs can provide comparable benefits to those of in-person intervention while allowing for more customization and flexibility to accommodate the patient’s needs and schedules [89]. The COVID-19 pandemic led to an urgent demand for innovation in the use of remote procedures in all sectors. In healthcare, many governments and institutions adopted a telemedicine system approach to patient care. Similarly, the use of mobile applications and health-related electronic devices, including digital therapeutics (software-driven therapeutic approaches for the diagnosis, prognosis, and treatment of pathological conditions) have exponentially increased as a response to the urgent need for patient-centered interventions [90,91,92]. Hence, computerized cognitive stimulation has been presented as a potentially valuable tool that allows the patient to undertake the training when it best suits their own agenda while allowing the healthcare professional to monitor the patient’s progress. Furthermore, the computerization of the intervention allows greater sensitivity to individual differences and adaptability to the patient’s performance, without being dependent on the material and personal resources of the health center.

Taking into consideration the preceding evidence, cognitive stimulation appears to be a versatile therapeutic tool with great potential [54,73,93,94]. However, despite the promising outcomes, standardized protocols have not been established yet, and possible side effects, as well as the generalization to other areas of daily functioning remain unknown. In the present study, we aimed to evaluate the safety and feasibility of a computerized cognitive stimulation intervention in breast cancer patients and its potential benefit on cognition and psychological well-being.

## 2. Materials and Methods

### 2.1. Study Design

A Phase I/II clinical trial was carried out to ascertain the intervention’s safety and to establish the maximum time of computerized cognitive stimulation that could be tolerated per session without producing unfavorable effects. The Section 2.5 below provides a detailed description of the safety measures used to monitor the participants and identify any potential adverse effects of the intervention. The protocol was approved by the participating hospital’s local Ethics Committee and by the Ethics Committee of Universidad Nebrija. Before being enrolled in the study, each participant received a thorough explanation and gave signed informed permission. The protocol was registered in ClinicalTrials.gov with the code NCT05409248.

### 2.2. Participants

All participants were recruited from the Breast Cancer Unit of the Hospital Universitario de La Ribera (Spain) using a non-probability criterion sampling method based on predetermined criteria relevant to the study objectives. The eligibility criteria were as follows: a confirmed diagnosis of breast tumor by histology; completion of chemotherapy treatment at least one year prior to the study; self-reported cognitive complaints; currently undergoing hormone treatment with tamoxifen or aromatase inhibitors; and being 18 years of age or older. Individuals with metastases or brain tumors, significant visual or movement impairment, or any other medical, mental, or neurological condition were excluded from participation in the study, as were those with alcohol or drug misuse or dependency, or who were currently receiving a cognitive intervention.

The final sample included ten breast cancer patients from a rural area, with a mean age of 51 years (range 35–67). In terms of education, 40% hold a college education diploma, 20% graduated high school, and 40% completed general education development or similar. Regarding their marital status, 80% were married, 10% were single, and 10% were widow. As for their employment situation, 30% were on medical leave, 30% remained active workforce, 30% were retired, and 10% were unemployed. Before intervention, a group of three participants, ranging in age from 37 to 64 years (mean age of 52 years), were recruited to establish the maximum tolerable dose of training per session, as outlined in the Procedure section. The group consisted of two married individuals with a general education background who were actively employed and one divorced individual with a high school education who was retired. Participant characteristics are presented in Table 1.

### 2.3. Cognitive Stimulation Intervention

**Figure 1:**
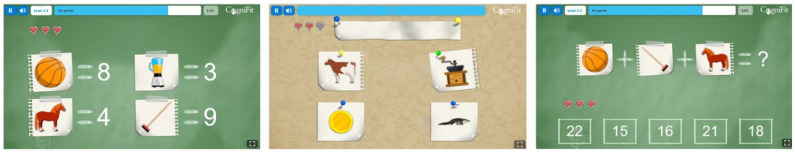
Example of a cognitive stimulation activity. This specific activity is designed to improve attention, working memory, and recognition. The task entails memorizing an image and its associated value (**left picture**); remembering a string of pictures and their locations (**middle picture**); and performing a mathematical calculation based on the values that were initially assigned to each image (**right picture**).

The computerized cognitive stimulation intervention was conducted using the CogniFit cognitive training platform (CogniFit Inc., San Francisco, CA, USA). The gamified cognitive stimulation activities (see Figure 1) selected were developed to improve specific cognitive functions, mainly executive functions, memory, and concentration. The computerized system operates using a patented Individualized Training System™ (ITS) software that enables all tasks to be customized to each user’s unique cognitive profile. This means that the difficulty of each activity or task is automatically aligned with the user’s performance, always demanding an achievable cognitive effort, thus maintaining motivation and adherence to the intervention [95].

Before launching an activity for the first time, a brief tutorial aimed to verify that the instructions were understood was presented. If the tutorial was successfully completed, the activity would begin; otherwise, the instructions would be shown again, and the tutorial would restart. If at any time during a given activity the system would detect a high error rate, the instructions and the tutorial would be replayed. Each activity lasted for approximately 5 min. All activities had a pause button, and instructions could be accessed during the task if needed.

### 2.4. Procedure

Recruitment for the study was conducted by the medical team at the Breast Cancer Unit of the Hospital Universitario de La Ribera. Patients who attended follow-up consultations and met the inclusion criteria were informed of the study by their physician. Interested patients were then referred to the clinical psychologist, who scheduled an in-person appointment to provide detailed information about the study and obtain their signed informed consent. A total of 22 female patients who met the inclusion criteria were cited and 13 of them (59.1%) voluntarily enrolled in the study. Three patients withdrew from the study. One patient abandoned on the second day due to time constraints; another patient left the trial on the fourth day because of medical complications; one final patient left on the eighth day due to personal circumstances.

**Figure 2:**
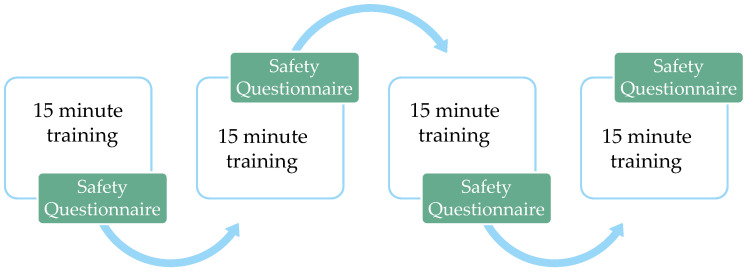
Timeline representation of Phase I.

A Phase I with a dose-escalation 3 + 3 design was conducted to determine the maximum tolerable time of cognitive stimulation without the presence of undesired events. The protocol consisted of performing the cognitive stimulation activities on a smartphone in various cycles of 15 min. Each 15-min cycle included three activities of 5 min each. At the end of each cycle of activities, a safety questionnaire was administered (see Figure 2). The questionnaire consisted of a 0-to-10 Likert-like scale to indicate the current level of fatigue, a Yes/No question regarding the presence of adverse effects, and a Yes/No question regarding the presence of unpleasant experiences (see Section 2.5). If the patient reported extreme fatigue levels (8 or higher on the Likert-like scale) or answered Yes to any of the two safety questions, the procedure would be stopped, and a structured interview would be conducted (see Section 2.5) to identify the extent of side effects. If no high fatigue or negative effects were reported, the procedure would continue with another 15-min cycle of cognitive stimulation, immediately followed by the same safety questionnaire. The maximum tolerated time of cognitive stimulation is determined according to the safety protocol [96]. If the protocol was stopped in the same cycle in at least 2/3 of patients, the cognitive stimulation session time would be set to that of the previous cycle (namely, of 2/3 of patients would report high fatigue after the 4th 15-min cycle, the tolerated session time would be set to 45 min, corresponding to 3 full cycles). In case convergent responses are not obtained from 2/3 of the patients, three additional participants would be administered with the dose of cognitive stimulation time reported in the previous cohort. If no patients report negative effects, the dose will escalate. If at least 1/3 of the patients report negative effects, then the previous dose would be considered the maximum tolerable cognitive stimulation time. The entire process was carried out individually in the hospital facilities and was supervised by a clinical psychologist.

**Figure 3:**
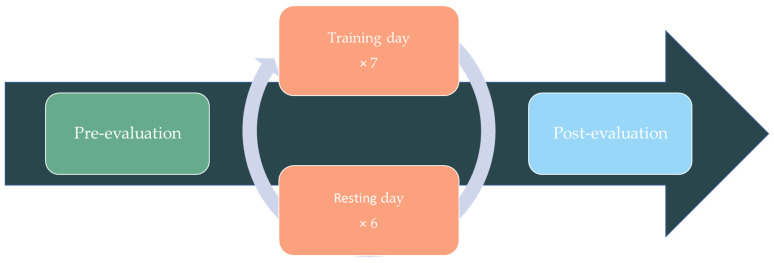
Timeline representation of Phase II.

After establishing the maximum tolerated time, a Phase II was conducted to explore the intervention’s efficacy and safety in the absence of adverse effects. A 15-day cognitive stimulation intervention with a pre- and post-evaluation was carried out (see Figure 3). On day 1 and day 15, a complete assessment of cognitive performance, daily functionality, emotional state, and quality of life was performed (see Section 2.5), with an approximate duration of 1 h. Cognitive stimulation sessions took place from the 2nd to the 14th day, with a rest day between sessions. Each intervention session (days 2, 4, 6, 8, 10, 12 and 14) comprised a total of nine activities with an approximate duration per activity of 5 min, being estimated 45 min per session (in accordance with the results of the Phase I trial; see below for details). The participants’ task was to complete all activities and, immediately after finishing, respond to the safety questionnaire regarding the level of fatigue and potential unwanted experiences encountered because of the intervention. If any participant reported extreme fatigue levels (being equal to or higher than 8 out of 10), or adverse effects, the clinical psychologist of the team would proceed to administer the structured interview (see Section 2.5). The activities of each session and their order of presentation were randomized. The activities were customized and self-adjusted based on each participant’s cognitive profile and performance. As a result, completing the tasks always required a cognitive effort, which boosted motivation and engagement in the intervention [95,97,98]. The intervention was carried out remotely by the participants from their smartphones using the CogniFit mobile application [99]. The entire process was monitored online by a clinical psychologist, with daily contact after the completion of the activities.

### 2.5. Assessment Measures

Safety measures. In both phases, a Safety Questionnaire was used immediately after finishing each intervention session. The questionnaire consisted of three items. On the first item, patients were asked to assess their current level of fatigue on a 0–10 Likert-like scale (0 = not at all fatigued; 10 = very fatigued). On the second item, patients were asked to indicate whether they had experienced any side effect associated to the intervention. Similarly, the third item asked about the feeling of any undesirable experience associated to the intervention. In case a patient reported adverse symptoms, a clinical psychologist administered a structured interview adapted from the Patient-Reported Adverse Drug Event Questionnaire [100].

Assessment instruments. In Phase II, a pre- and post-intervention evaluation was conducted. A total of seven evaluation instruments were used to assess the effectiveness of the cognitive stimulation program. As primary outcome, the following instruments were included for assessing the cognitive, physical, and functional impact of cancer: the mini-Mental Adjustment to Cancer Scale (Mini-MAC), the Functionality Assessment Instrument in Cancer Treatment–Cognitive Function (FACT-COG), and the Brief Fatigue Inventory (BFI). The Mini-MAC examines five particular coping techniques for cancer: anxiousness and preoccupations, cognitive disengagement, tenacity, powerlessness feeling, and fatalistic thinking [101]. The FACT-COG assesses the patient’s self-perspective of their cognitive ability, cognitive dysfunction in everyday functioning, and overall quality of life [102]. The BFI examines the influence and severity of cancer-related tiredness [103]. Secondary outcome measures were aimed at assessing cognitive performance, emotional anguish, and general well-being. These instruments were the following: the Cognitive Assessment for Chemo Fog Research (CAB-CF); the Beck Depression Inventory (BDI-II); the State-Trait Anxiety Inventory (STAI); and the Measuring Quality of Life|The World Health Organization–abridged version (WHOQOL-BREF). The CAB-CF is a neuropsychological evaluation battery that assesses cognitive domains such as memory, attention, reasoning, perception, and coordination while also providing a complete cognitive screening and evaluating the risk index of suffering cancer-related cognitive impairment (CogniFit Inc., San Francisco, CA, USA). The BDI-II provides information on the presence and severity of depressive symptomatology [104]. For the STAI, only the state anxiety dimension was employed to detect the existence of current anxious symptomatology [105]. The WHOQOL-BREF provides a profile of self-perceived quality of life [106].

Here we used the total global score for each assessment instrument, following the specific instructions provided in each test or tool. For instance, the Mini-MAC questionnaire has 29 items, each with a 4-point Likert response scale, yielding scores ranging from 29 to 116. A higher score implies the use of more coping strategies. Similarly, the FACT-COG has 37 items, each with a 4-point Likert response scale, yielding scores ranging from 0 to 148, with a higher score indicating better cognitive function. The BFI comprises one dichotomous-response item and nine items with a response range of 1–10, with scores ranging from 9 to 99, and where higher scores indicate higher fatigue levels. We only considered the state sub-scale of STAI, which has 20 items with four response options, yielding scores ranging from 0 to 60, with a higher score indicating more anxious symptoms. The BDI-II has 21 items with four response options, and scores range from 0 to 63, with higher scores indicating more significant depressive symptoms. WHOQOL-BREF, on the other hand, has 28 quantitative questions, and scores range from 28 to 140, with higher scores indicating better quality of life. The CAB-CF is made up of a global score ranging from 0 to 800 obtained through 17 activities.

### 2.6. Data Analyses

All data collected were processed using RStudio [107], and analyzed with JASP (Version 0.17) [108]. Descriptive statistics were conducted followed by paired *t*-tests to determine statistically significant differences between pre- and post-intervention scores on each of the assessment instruments. Furthermore, repeated measures ANOVAs were performed across two time points (pre-intervention and post-intervention) to evaluate intervention effectiveness and assess possible interactions with independent variables such as participant age or time since chemotherapy completion. The statistical significance differentiation criterion was set at *p* < 0.05.

## 3. Results

Phase I. The phase concluded with the first cohort of three participants, all of whom reported fatigue levels at or above 8 on the Safety Questionnaire after the fourth cycle of activities. No participants reported negative effects or undesired experiences derived from the procedure. Since all three patients reported fatigue after 60 min of cognitive stimulation (see Table 2), the maximum time allocated for Phase II was 45 min (namely, the time corresponding to the previous activity cycle), varying between 40 and 45 min depending on each participant’s game objective achievements. Phase I participants were not the same individuals as those included in Phase II, although the eligible criteria were the same.

Phase II. No participant reported negative effects or extreme fatigue on the Safety Questionnaire in any of the intervention sessions. Descriptive analyses of pre- and post-test scores are displayed in Table 3.

Data from the pre- vs. post-intervention were analyzed using paired t-test comparisons. The normality assumption check using the Shapiro–Wilk test yielded non-significant *p*-values (*p*-values ranged from 0.33 to 0.94), indicating that the pairwise differences are normally distributed, and thus the assumption of normality is not violated. As shown in Table 4, results from the Mini-MAC scale, the CAB-CF, and the BDI-II showed significant differences between pre- and post- intervention. The rest of measures (FACT-COG, BFI, STAI, and WHOQOL-BREF) did not show any significant differences.

To address the potential influence of age and time since chemotherapy completion, we conducted a 7 (Evaluation Test: Mini-MAC, FACT-COG, BFI, CAB-CF, BDI-II, STAI, WHOQOL-BREF) × 2 (Evaluation Phase: pre-treatment and post-treatment) repeated measures ANOVA that included age at the beginning of the study and lapsed time between the end of chemo treatment and the beginning of the study as covariates. No significant effect was found for evaluation phase F(1, 7) = 1.25, *p* = 0.3, η^2^_p_ = 0.001. Furthermore, sphericity assumptions were violated evaluation test; thus, Greenhouse–Geisser correction was applied. A significant main effect of evaluation test was found F(1.23, 6.00) = 15.14, *p* < 0.003, η^2^_p_ = 0.575). Post hoc analyses showed significant differences between our seven evaluation test levels (see Supplementary Material Table S1 for post hoc test comparison). Moreover, evaluation test did not correlate with any of our covariate measures F(1.23, 6.00) = 1.94, *p* = 0.201, η^2^_p_ = 0.074 for age at the beginning of the study and F(1.23, 6.00) = 0.34, *p* = 0.621, η^2^_p_ = 0.013 for lapsed time between the end of chemo treatment and the beginning of the study; note that as sphericity was violated Greenhouse–Geisser correction was applied.

## 4. Discussion

The present study aimed to investigate the feasibility of a self-administered computerized cognitive stimulation intervention for cancer survivors with cognitive impairment. The participants, who had overcome breast cancer, received a 45-min multi-session cognitive stimulation intervention remotely from their personal electronic devices while being monitored by a healthcare professional. The pre- and post-evaluation revealed that the intervention was effective and well-tolerated by patients, with no adverse effects reported. The results showed significant improvements in cognitive performance, attitude toward cancer-related impairment, and mood after only seven intervention sessions. Other measures such as the fatigue level, the perceived cognitive impairment, the daily functionality, the level of anxiousness, and the quality of life also improved numerically, even though they did not reach statistical significance compared to the baseline.

Our study’s findings align with the existing research on the advantage of cognitive training, which indicates that this intervention does not have any detrimental effects [109], and can enhance the cognitive function and reduce depressive symptomatology [110,111]. In addition to the cognitive and mood improvements we observed, we also found a significant enhancement in coping strategies. Depression and cognitive performance have a well-established link [112]. Depression is a clinical condition that can severely affect cognitive abilities, particularly those that rely on attention, memory, and executive functions [113]. Individuals with depression may struggle with concentration, recall, decision-making, and problem-solving, which can interfere with their daily life and contribute to a further decline in mood, leading to a vicious cycle of decline [114]. Cognitive stimulation interventions have been shown to be effective in improving both cognitive function and mood. The relationship between these constructs is not surprising, given their interdependence. Several studies have demonstrated that cognitive stimulation can improve mood and daily functioning in individuals with major depressive disorder [76,115]. By enhancing cognitive abilities, individuals may feel more confident in their ability to perform tasks, resulting in a more positive mood, and an increased ability to cope with daily stressors [116]. Therefore, the positive effects of cognitive stimulation interventions observed in the BDI-II, CAB-CF, and Mini-MAC measurements in our study can be attributed to the improvement of cognitive abilities found in individuals with depression. The results support the relevance of incorporating cognitive training as part of the management of CRCI, as it can improve both cognitive function and mood.

Regarding quality of life, the benefits of cognitive stimulation interventions remain uncertain. While some studies have shown improvements in quality of life in individuals with clinical insomnia after just seven sessions [74], studies in healthy elderly populations have not found significant differences in quality of life after either short-term (24 sessions performed twice a week) [117] or long-term (over 12 months) [118] cognitive stimulation programs that use electronic devices. Despite finding contradictory results in the literature, those studies that have included more than one training session per week, as in our case, have consistently reported improvements [109]. Similarly, most randomized controlled trials have reported only small benefits or minor clinical improvement on cognition, mood, behavior, and daily functioning [109,111], which could explain the lack of significant differences we found in our study regarding the FACT-COG, BFI, STAI, and WHOQOL-BREF assessments. Qualitative studies conducted in healthy elderly populations or those with mild-to-moderate cognitive impairment have also shown that cognitive training can enhance coping strategies [119], explaining the enhancement of those strategies as reported by the current participants in the Mini-MAC. Previous studies have also shown that computerized cognitive training in breast cancer survivors can lead to subjective improvements and transfer to behavior and daily functioning [120,121,122].

The discrepancies we encounter among the studies in the effectiveness of cognitive training interventions may be attributed to several factors, including the specific activities and cognitive domains targeted by the training, as well as the number and duration of the training sessions [123]. For example, previous studies have focused on fewer cognitive domains, such as executive functions [120], memory and processing speed [124], or memory and attention [125], whereas our training program involved a variety of activities targeting different domains. The inconsistent findings across studies on the effectiveness of cognitive stimulation interventions could be attributed to various factors, such as the absence of a concise definition of cognitive stimulation [110] and the lack of a standardized protocol for its implementation [126]. Nonetheless, studies in the literature have found consistent benefits in cognitive stimulation programs that involve at least two sessions per week, each lasting more than 30 min, regardless of the duration of the program. This consistency of results is based on the concept of neuronal plasticity, which implies that regular mental exercises are necessary for neuronal enhancement. However, it is also possible that the improvements reported in some studies are more closely linked to the underlying pathology than to the benefits of cognitive stimulation itself, which highlights the need for further research in this area.

The clinical implications of this study suggest that a self-administered computerized cognitive stimulation intervention can be an effective and well-tolerated intervention for cancer survivors with cognitive impairment, specifically those who have overcome breast cancer. This intervention can lead to significant improvements in cognitive performance, attitude toward cancer-related impairment, mood, and coping strategies, which can improve daily functioning and overall well-being. However, further research is needed to explore the impact of cognitive stimulation interventions on quality of life and to determine the optimal frequency and duration of the intervention to maximize its benefits.

### Limitations

Although our results shed new light on the potential usefulness of cognitive training and its benefits in oncology population, it is important to acknowledge the limitations that may affect the interpretation of the data. Our study is a preliminary exploratory investigation aimed at determining the suitability, potential benefits, and usefulness of a home-based intervention utilizing gamified computerized cognitive stimulation software. The absence of a control group prevents us from determining whether the effects obtained are due to the simple fact of being involved in a clinical intervention, or whether they are in fact due to cognitive stimulation. Moreover, the sample’s modest size and variability, and the low number of sessions that were initially used in this feasibility pre-post study also hinder the generalization of our results. Nonetheless, the results of our Phase I/II trial already suggest that the use of a computerized cognitive training has the potential to enhance cognitive functioning in cancer survivors, specifically improving cognitive performance in memory, attention, perception and reasoning domains, use of coping strategies, and mood without any significant side effect, undesirable experience, or extreme fatigue.

## 5. Conclusions

This is one of the few studies that aims at assessing the safety and efficacy of home-based computerized cognitive stimulation as a clinical intervention to reduce the cancer-related cognitive impairment, broadening the applicability of digital therapeutics toward the treatment of cognitive decline resulting from chemotherapeutic agents, and yielding encouraging results for this population. Our results yield some encouraging improvements not only in cognitive performance and depressive symptoms but also in the use of coping strategies. These findings have significant practical implications for the daily life of cancer survivors. The improvements in coping strategies can lead to increased capacity to manage strong impulses and emotions, improved self-esteem, increased confidence in their own strengths and abilities, greater emotional regulation, better resilience, and promoting healthy self-care [127,128,129,130]. Future studies should further explore such promising benefits by exploring the effectivity in larger samples and providing a longer-term view by conducting randomized control trials.

## Figures and Tables

**Table 1:** Participant characteristics.

Age	Phase Participation	Marital Status	Education	Employment
64	Phase I	Divorced	High School	Retired
54	Phase I	Married	General education	Active worker
37	Phase I	Married	General education	Active worker
65	Phase II	Married	General education	Retired
47	Phase II	Married	Bachelor’s Degree	Active worker
47	Phase II	Single	General education	Sick leave
38	Phase II	Married	High School	Unemployed
48	Phase II	Married	Bachelor’s Degree	Active worker
67	Phase II	Widow	General education	Retired
66	Phase II	Married	High School	Retired
42	Phase II	Married	General education	Active worker
54	Phase II	Married	General education	Sick leave
33	Phase II	Married	Bachelor’s Degree	Sick leave

**Table 2:** Fatigue scores reported after each cycle of activities in Phase I.

	Cycle-I	Cycle-II	Cycle-III	Cycle-IV
Participant 1	2	3	5	9
Participant 2	1	4	7	9
Participant 3	3	5	7	10

**Table 3:** Statistic descriptive at pre- and post-evaluation for all assessment measures.

	Mean (SD) Pre-Evaluation	Mean (SD) Post-Evaluation
Mini-MAC	84.50 (6.11)	86.90 (6.17)
FACT-COG	80.30 (25.31)	86.10 (38.15)
BFI	21.00 (12.98)	17.60 (11.74)
STAI	45.90 (5.08)	43.40 (5.33)
BDI-II	36.20 (6.77)	31.80 (6.90)
WHOQOL-BREF	90.80 (9.41)	91.40 (8.93)
CAB-CF	325.70 (123.37)	567.20 (96.38)

Note that for certain assessment instruments (BFI, BDI-II, and STAI), a lower score reflects an improvement in the evaluated domain.

**Table 4:** Paired samples *t*-test for assessment measures pre- and post- cognitive stimulation intervention.

					95% CI for Mean Difference		
	t	*p*	Mean Difference	SE Difference	Lower	Upper	Cohen’s d
Mini-MAC	2.44	0.037 *	−2.40	0.98	−4.61	−0.18	−0.77
FACT-COG	0.86	0.411	−5.80	6.72	−21.00	9.40	−0.27
BFI	1.15	0.278	3.40	2.94	−3.26	10.06	0.36
STAI	1.95	0.082	2.50	1.27	−0.38	5.38	0.62
BDI-II	2.52	0.032 *	4.40	1.74	0.46	8.33	0.80
WHOQOL-BREF	0.21	0.838	−0.60	2.84	−7.04	5.84	−0.06
CAB-CF	7.11	<0.001 ***	−241.50	33.95	−318.30	−164.69	−2.24

The scores reflect the mean scores obtained by the participants in each of the assessment instruments at the pre-evaluation (day 1) and post-evaluation (day 15). All degrees of freedom were 9. * *p* < 0.05; *** *p* < 0.001.

## Data Availability

The data supporting the results of the present study are not publicly available due to the data protection regulation of the Spanish public health system.

## Supplementary Materials

The following supporting information can be downloaded at: https://www.mdpi.com/article/10.3390/ijerph20064953/s1, Table S1: Within subject effect Repeated measures ANOVA.
