# Supplementary material for: Effectiveness of a Computerized Home-Based Cognitive Stimulation Program for Treating Cancer-Related Cognitive Impairment
Source: Int J Environ Res Public Health. 2023 Mar 11;20(6):4953. doi: 10.3390/ijerph20064953 (PMC10049401; doi:10.3390/ijerph20064953)
Supplement: Supplementary file 1 [file ijerph-20-04953-s001.zip › ijerph-2194932-supplementary.pdf]

Supplementary Material 1. Additional analyses results.

Table S1. Within subject effect Repeated measures ANOVA.

Cases	Sphericity Correction	Sum of Squares	df	Mean Square	F	p	η^2
Pre-Post	None	654.57	1.000	654.57	1.254	0.300	0.001
Pre-Post ✻ Lapsed time between the end of chemo treatment and the beginning of the study	None	2915.81	1.000	2915.81	5.586	0.050	0.006
Pre-Post ✻ Age at the beginning of the study	None	1.02	1.000	1.02	0.002	0.966	< 0.001
Residuals	None	3653.83	7.000	521.97			
Test	None	267305.25[a]	6.000[a]	44550.87	15.142[a]	< .001[a]	0.575
	Greenhouse-Geisser	267305.25	1.227	217802.15	15.142	0.003	0.575
Test ✻ Lapsed time between the end of chemo treatment and the beginning of the study	None	5954.60[a]	6.000[a]	992.43	0.337[a]	0.913[a]	0.013
	Greenhouse-Geisser	5954.60	1.227	4851.85	0.337	0.621	0.013
Test ✻ Age at the beginning of the study	None	34269.72[a]	6.000[a]	5711.62	1.941[a]	0.096[a]	0.074
	Greenhouse-Geisser	34269.72	1.227	27923.20	1.941	0.201	0.074
Residuals	None	123568.63	42.000	2942.11			
	Greenhouse-Geisser	123568.63	8.591	14383.51			

Note. Sphericity corrections not available for factors with 2 levels.

Note. Type III Sum of Squares.

[a] Mauchly's test of sphericity indicates that the assumption of sphericity is violated ($p < .05$).
